# The Role of Uron and Chlorobenzene Derivatives, as Potential Endocrine Disrupting Compounds, in the Secretion of ACTH and PRL

**DOI:** 10.1155/2018/7493418

**Published:** 2018-05-29

**Authors:** Krisztian Sepp, Anna M. Laszlo, Zsolt Molnar, Andrea Serester, Tunde Alapi, Marta Galfi, Zsuzsanna Valkusz, Marianna Radacs

**Affiliations:** ^1^First Department of Medicine, Faculty of Medicine, University of Szeged, Szeged, Hungary; ^2^Department of Biometrics and Agricultural Informatics, Faculty of Horticultural Science, Szent István University, Budapest, Hungary; ^3^Department of Environmental Biology and Education, Institute of Environmental and Technological Sciences, Juhász Gyula Faculty of Education, University of Szeged, Szeged, Hungary; ^4^Department of Inorganic and Analytical Chemistry, Faculty of Science and Informatics, University of Szeged, Szeged, Hungary

## Abstract

Uron herbicides polluting the environment represent a serious concern for environmental health and may be regarded as endocrine-disrupting compounds (EDCs), which influence the regulation of human homeostasis. We aimed to investigate the effect of EDC urons (phenuron: PU, monuron: MU, and diuron: DU) and chlorobenzenes on the basal release of the adrenocorticotropic hormone (ACTH), which is a part of the adenohypophysis-adrenocortical axis. Hormone secretion in the presence of EDC was studied in two cell types: normal adenohypophysis cells (AdH) and cells of prolactinomas (PRLOMA). PRLOMA was induced in female Wistar rats by subcutaneously injecting them with estrone acetate for 6 months. AdH and PRLOMA were separated from treated and untreated experimental animals, dissociated enzymatically and mechanically in order to create monolayer cell cultures, which served as an experimental *in vitro* model. We investigated the effects of ED agents separately and in combination on ACTH and prolactin (PRL) release through the hypophyseal-adrenal axis. Hormone determination was carried out by the luminescent immunoassay and the radioimmunoassay methods. Our results showed that (1) uron agents separately did not change ACTH and PRL release in AdH culture; (2) ACTH secretion in arginine vasopressin- (AVP-) activated AdH cells was significantly increased by EDC treatment; (3) ED agents increased the basal hormone release (ACTH, PRL) in PRLOMA cells; and (4) EDC exposure increased ACTH release in AVP-activated PRLOMA cells. We conclude that the herbicides PU, MU, and DU carry EDC effects and show human toxicity potential.

## 1. Introduction

Chemical agents (e.g., halogenated hydrocarbons and uron herbicides [1–3]) which pollute the environment represent a serious concern for environmental health [4, 5] and may be regarded as endocrine-disrupting compounds (EDCs), which influence the regulation of human homeostasis [6]. They may change the potential [7, 8] and capacity [9, 10] of the psycho-neuro-endocrine-immune regulation network and may bring about disturbances in the regulatory process [11, 12] which result in serious homeostatic alteration even in healthy individuals [13, 14]. Human adaptation patterns induced by environmental burdens are obviously modified when the exposition reaches an individual who suffers from functional and/or structural disorders (illnesses) [15, 16]. An open dynamic system which forms a unit with its environment, for example, a living human organism, can stabilize its equilibrium processes as determined by the direct environmental conditions (attraction range) [17] defined by its genetic and functional adaptation potential (algorithmic networks characterizing the local properties of the living system [18]). In human homeostasis, hormones are the creative elements of the neuroendocrine regulation [19, 20]. Human neuroendocrine regulation can be interpreted as a network of open, dynamic biological systems [21] in the outlined context. Biological cycles that can be described with the “*AND*” function are those essential for life (e.g., human hypothalamus-adenohypophysis-adrenal cortex axis functional disorder) [22]. The “*OR*” function-related control systems are not essential at the organizational level of the given individual (e.g., PRL); life functions can be maintained in their absence. The disturbance of the healthy (control) processes of the “*OR*” cycle will affect the “*AND*” cycles. Chronic changing of the “*OR*” cycle may lead to structural disturbance, for example, cellular proliferation, which is sustained by continuous feedback information [23].

Herbicides [24] and halogenated hydrocarbons [25] are widespread substances that have an EDC effect. Phenuron (PU), monuron (MU), and diuron (DU) compounds may be viewed as a halogenated homologous series of herbicides [26]. Chlorobenzenes (ClB) represent a halogenated aromatic hydrocarbon group, of which 1,4-dichlorobenzene (dClB) is known as an international reference compound due to its ecotoxicological and human toxicity potential [27]. It is a primary question from the point of view of medical practice, whether the cellular follow-up of ACTH regulation is a suitable test system for studying the effect of the environmental pollutants. Any exposure can be interpreted as a stressor in the human neuroendocrinium [28]. The outlined circuit (in mathematical terms: attractor) maintains processes indispensable in human homeostasis, since there is no life without adrenocortical hormones. The regulatory disturbances (e.g., feedback disturbances) may become causative factors, for example, they may lead to benign cell proliferation. The most commonly occurring human adenohypophysis cell proliferation is prolactinoma, which generates prolactin overproduction. In the development of the tumor, a significant pathophysiological role is attributed to the estrogenic effect [29].

When investigating the neuroendocrine aspects of ED agents (e.g., uron herbicides and ClB), the experimental layout is based on preliminary experiments (dose and time kinetics) [30] and the already proven physical, chemical, and biological effects of the compounds. Urons are substituted phenylureas of high chemical stability, which are used in agriculture as photosynthesis-inhibiting herbicides [31]. Due to their long chemical half-life (a few months to one year in soil, 2–6 weeks in water), the food chain can be severely affected [32, 33]. In humans, low to moderate toxicity is associated with spleen and liver involvement, whereas carcinogenicity [34] has also been demonstrated, and in the case of diurons, antiandrogenic properties have been described as well [35, 36]. Chlorobenzenes are also highly persistent chlorine-substituted aromatic hydrocarbons forming a homologous series [2, 37, 38]. Toxicity studies report liver and kidney impairment, but their roles as carcinogenic (breast, liver, and kidney) agents are also known [39, 40]. As EDC effect, the alterations of thyroid hormone synthesis and androgenic functions have been discovered [41, 42]. The human toxicity potential (HTP) is an internationally standardized impact category set in LCA (life cycle analysis) standards, used to express various environmental effects [43], in which the effects of environmental 1,4-dichlorobenzene on human health are the benchmark. The determination and relation of ecotoxicity potential (ETP) to dClB effects are performed in a similar fashion.

Our aims were to investigate the effect of ED compounds ((PU, MU, and DU) and dClB and 1,2,4-trichlorobenzene + hexachlorobenzene (chlorobenzene mixture, mClB)) on the basal release of the ACTH involved in the functioning of the adenohypophyseal-adrenocortical axis. This work focused on hormone secretion in the presence of EDC in two cell types: normal adenohypophysis (AdH) cells and prolactinoma (PRLOMA) cells. In this regard, the question was, Can EDC modify the feedback mechanism of ACTH release governed by arginine vasopressin (AVP) and corticosterone (B) (“*AND*” cycles)? Observing EDC effects on PRL hormone secretion was also a goal in this research (“*OR*” cycle).

## 2. Materials and Methods

### 2.1. Experimental Animals

Certified healthy female rats were used in our experiments (Wistar strains weighing 120–250 g, 4–6 weeks old at the onset of the study) (Charles River, Isaszeg, Hungary). During the experimental period, animals were kept in a controlled (55–65% relative humidity, 22 ± 2°C air temperature), automated diurnal environment (12 h daytime, 12 h night illumination cycles) in 32 × 40 × 18 cm cages (5 animals/cage). The diet required for experimental animals (CRLT/N, Charles River, Hungary) and drinking water were available ad libitum. The animals involved in the study were treated in accordance with Gov. Ordinance Number 40/2013 (II. 14.) on animal experiments.

### 2.2. Induced Prolactinoma and *In Vitro* Experimental Models

The PRLOMA models were made from Wistar rats (♀, *n* = 20) which were subcutaneously injected with estrone acetate for 6 months (CAS registration number 901-93-9, Sigma, Germany, 150 *μ*g/kg/week) [44]. After the pentobarbital (4.5 mg/kg, Nembutal, Abbott, USA) anesthesia, the animals were decapitated and AdH was separated; the tissue was enzymatically (trypsin: Sigma, Germany, 0.2% for 30 min; collagenase: Sigma, Germany, 30 *μ*g/ml for 40 min; dispase: Sigma, Germany, 50 *μ*g/ml for 40 min; phosphate-buffered saline (PBS-A) was used for the solutions, temperature: 37°C) and mechanically (83 *μ*m and 48 *μ*m pore size nylon blutex filter) dissociated. The cell viability was ≥95% (trypan blue staining). The cells were suspended in the following medium: Dulbecco's Modified Essential Medium (DMEM, Sigma, Germany) + 20% fetal calf serum (FCS, Sigma, Germany) + 1.0 IU/ml penicillin + streptomycin (Sigma, Germany). Then, they were placed into surface-treated (5% collagen) 24-well plastic culture vessels (Nunc, Germany) and put in a thermostat (temperature = 37°C, pCO_2_: 5%). Cell cultures were washed every 3 days after adherence until they became confluent.

**Figure 1:**
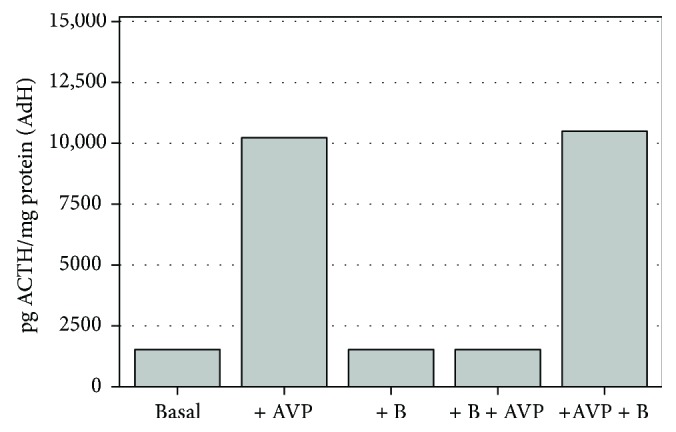
Functional assay for ACTH release in normal rat adenohypophysis cultures, *in vitro* (*n* = 8–12 in each group); mean level ± SEM (pg ACTH/mg protein): AVP significantly increases (*p* < 0.001), whereas corticosterone alone does not alter ACTH release when compared to control; preincubation with +B inhibited AVP activation (basal: control; AVP: 8-arginine vasopressin; B: corticosterone).

**Figure 2:**
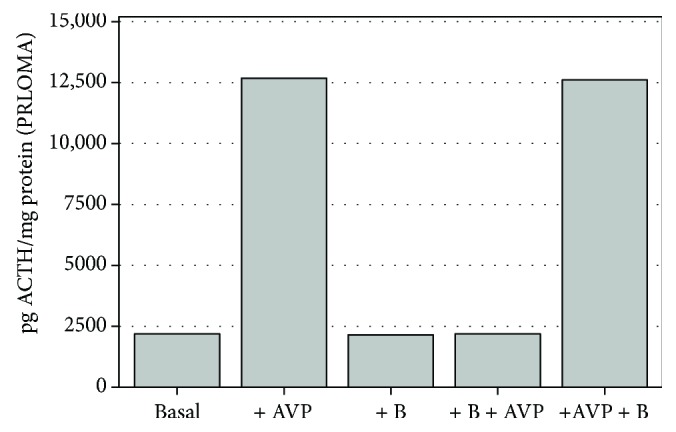
Functional assay for ACTH release in rat prolactinoma cultures (PRLOMA), *in vitro* (*n* = 11 − 12 in each group); mean level ± SEM (pg ACTH/mg protein): compared with normal AdH cells (Figure 1). ACTH release always shows a similarly significant (*p* < 0.001) increase in PRLOMA cells (basal: control; AVP: 8-arginine vasopressin; B: corticosterone).

Specific functional standardization for ACTH release was regulated by 1 *μ*g/ml corticosterone (B) and 10^−6^ M AVP treatments. The ACTH release cycle (“*AND*” cycles) was activated by AVP; this mechanism was inhibited by a 20-minute preincubation with B (Figures 1 and 2).

### 2.3. Experimental Protocol

Time and dose kinetic assays, determining the appropriate arrangements, were performed on standardized AdH and PRLOMA *in vitro* cultures. In the present study, AdH and PRLOMA cell cultures were first treated for 60 minutes with chlorobenzene (dClB = 0.1 ng/ml; chlorobenzene mix (mClB) = 0.1 ng/ml; hexachlorobenzene and 1,2,4-trichlorobenzene 1 : 1) and with urons (PU: 10^−6^ M, MU: 10^−6^ M, DU: 10^−6^ M). At the end of the EDC treatment, samples were obtained from the supernatant media of the cell cultures for the determination of ACTH and PRL hormones. When studying the ACTH-mediated role of EDC agents in the hypophysis/adrenal cortex regulation, the EDC agents were coadministered with (10^−6^ M) AVP and after the 60-minute treatment period, samples were taken from the supernatant media of both AdH and PRLOMA, in the regulation cycle of AVP/B feedback studies in Figures 1 and 2.

EDCs were added together with B. AVP was administered after a 20-minute pretreatment with EDC + B, and at the end of the treatment period, the supernatant media were sampled. In order to follow PRL hormone release, AdH and PRLOMA cell cultures were individually treated with ED agents for 60 minutes. Next, the supernatant media were used to measure PRL.

### 2.4. Hormone Assays

PRL assay was performed by radioimmunoassay from samples obtained according to the experimental protocol [44]. Determination of ACTH from samples was carried out by the luminescent immunoassay method, using the apparatus of the Endocrinology Unit, First Dept. of Internal Medicine, Faculty of Medicine, University of Szeged (Immulite 2000, Siemens Healthcare Diagnostic, Deerfield, IL, USA and DPC kit/L2KAC-02, Euro DPC Ltd., Glyn Rhonwy, United Kingdom). The protein content of the samples was determined using a modified Lowry method [45] and Pierce BCA Protein Assay Kit (Thermo Fisher Scientific Inc., Rockford, USA).

### 2.5. Statistical Analysis

Measurements (*n* = 8–12 per group on 24 lots: pooled samples on AdH cell cultures) of ACTH and PRL hormone release by disease (PRLOMA versus normal AdH) in various EDC groups (control, dClB, mClB, PU, MU, and DU) by regulation (basal, +AVP, +B, +B+AVP, and +AVP+B) were compared using mixed models on rats [46, 47]. The regulation cycle was verified in a mixed model for the comparison of the control groups of EDC for ACTH in the 5 regulation phases, using disease and regulation as fixed effects and random intercept for the lots. For ACTH data, a mixed model was applied with disease, EDC, and regulation (only basal, +AVP, and +B+AVP) as fixed factors and random intercept for the lots. For PRL measurements, a mixed model was applied with disease and EDC as fixed factors and random intercept for the lots for basal regulation data. In the analysis models, the reference group was the normal (healthy AdH), control (no EDC treatment), and basal (no regulation) group. Restricted maximum likelihood estimation and Kenward-Roger method for adjusting the degrees of freedom were applied in all models with unstructured covariance matrix. Pairwise comparisons were estimated by least squares means using Sidak *p* value adjustment. Model residuals were displayed on quantile-quantile plots to check normality assumptions. Statistical analyses were performed in SAS (version 9.3 SAS Institute Inc., Cary, NC, USA), where *p* values of <0.05 were considered to indicate statistical significance [48].

## 3. Results

**Figure 3:**
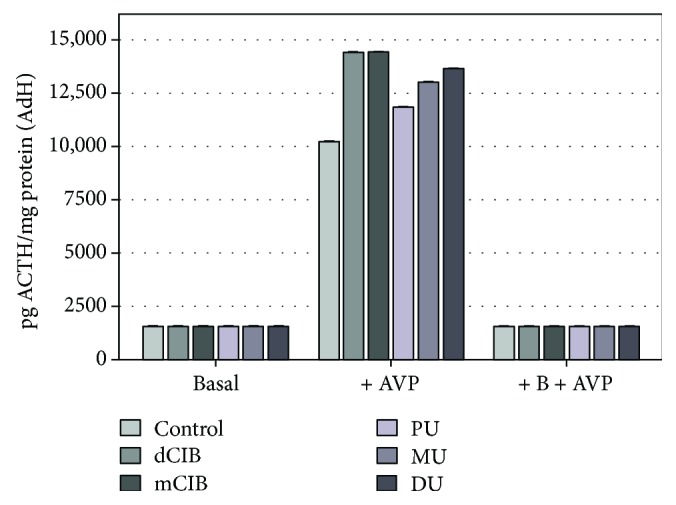
EDC effects in the regulation of ACTH release on normal rat AdH cultures, *in vitro*. Mean (ACTH level) ± SEM. The mean and SEM are calculated from *n* = 12. Abbreviations: B = corticosterone: 1 *μ*g/ml; AVP = 8-arginine vasopressin: 10^−6^ M, +B+AVP: in combination therapy B precedes AVP administration by 20 minutes; dClB = 1,4-dichlorobenzene: 0.1 ng/ml; mClB = chlorobenzene mix: 0.1 ng/ml; PU = phenuron: 10^−6^ M; MU = monuron: 10^−6^ M; DU = diuron: 10^−6^ M. All EDC groups differ significantly from the control (*p* < 0.001) for +AVP.

Figure 3 shows the effect of various ED compounds (dClB, mClB, PU, MU, and DU) on ACTH release in AdH cultures in the following cases: basal, AVP activated (+AVP), and the corticosterone-inhibited AVP activation (+B+AVP) in AdH cultures. It can be seen that ACTH release was not altered by ED agents (mean level ± SEM (pg ACTH/mg protein): dClB 1567.91 ± 3.09; mClB 1585.33 ± 2.72; PU 1533.67 ± 2.52; MU 1553.17 ± 3.40; and DU 1566.33 ± 2.30) compared to the control group (1528.25 ± 6.14 pg ACTH/mg protein). In the AVP-activated samples, ACTH release showed a significant increase compared to the control group for each EDC (mean level ± SEM (pg ACTH/mg protein): control 10,220.88 ± 20.36; dClB 14,430.08 ± 3.01; mClB 14,488.90 ± 3.57; PU 11,845.67 ± 7.02; MU 13,008.25 ± 10.18; and DU 13,658.75 ± 15.83). In the case of regulatory effect (+B+AVP-feedback), large deviations could not be detected in the presence of EDC (mean level ± SEM (pg ACTH/mg protein): control 1524.67 ± 3.46; dClB 1542.00 ± 1.22; mClB 1566.92 ± 2.40; PU 1540.08 ± 2.86; MU 1560.08 ± 1.88; and DU 1578.08 ± 1.26).

**Figure 4:**
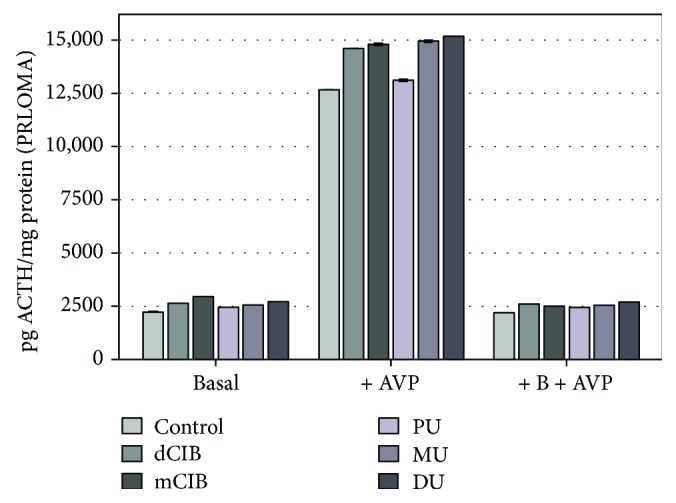
EDC effects in the regulation of ACTH release in rat PRLOMA cultures, *in vitro*. Mean (ACTH level) ± SEM. The mean and SEM are calculated from *n* = 12. Abbreviations: B = corticosterone: 1 *μ*g/ml; AVP = 8-arginine vasopressin: 10^−6^ M, +B+AVP: in combination therapy B precedes AVP by 20 minutes; dClB = 1,4-dichlorobenzene: 0.1 ng/ml; mClB = chlorobenzene mix: 0.1 ng/ml; PU = phenuron: 10^−6^ M; MU = monuron: 10^−6^ M; DU = diuron: 10^−6^ M. All EDC groups differ significantly from the control (*p* < 0.001) for basal, +AVP, and +B+AVP.

Figure 4 shows the effects of dClB, mClB, PU, MU, and DU on ACTH release in PRLOMA cultures in the following cases: basal, AVP activation (+AVP), and corticosterone-inhibited AVP activation (+B+AVP). It can be seen that ED agents modulate ACTH release compared to the control of the basal group (2193.64 ± 1.92 pg ACTH/mg protein): dClB: 2624.30 ± 7.60 pg ACTH/mg protein; mClB: 2956.08 ± 4.71 pg ACTH/mg protein; PU: 2427.33 ± 6.08 pg ACTH/mg protein; MU: 2535.17 ± 5.14 pg ACTH/mg protein; and DU: 2705.33 ± 4.63 pg ACTH/mg protein. AVP-activated ACTH release of PRLOMA cultures shows a significant increase for each ED compound used (mean level ± SEM (pg ACTH/mg protein): control 12,674.50 ± 7.23; dClB 14,620.58 ± 5.61; mClB 14,830.50 ± 7.42; PU 13,129.67 ± 6.06; MU 14,954.17 ± 11.22; and DU 15,197.58 ± 4.99). EDC effects were detected in the regulation model (+B+AVP-feedback) (mean level ± SEM (pg ACTH/mg protein): control 2195.50 ± 4.69; dClB 2579.42 ± 3.98; mClB 2513.00 ± 2.94; PU 2421.00 ± 2.28; MU 2553.83 ± 7.87; and DU 2690.42 ± 4.14).

**Figure 5:**
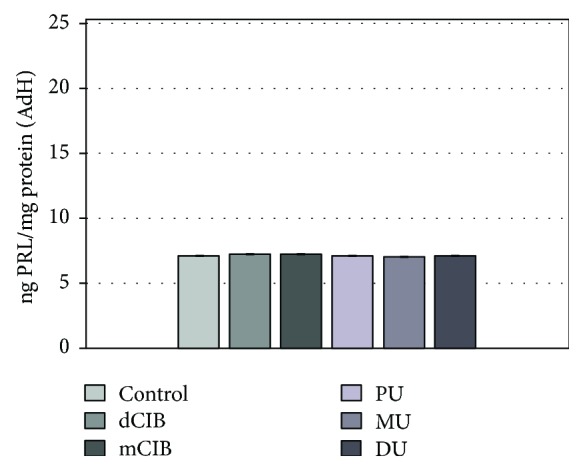
Effects of EDC on PRL release in normal rat AdH cultures, *in vitro*. Mean (PRL/prolactin/level) ± SEM. The mean and SEM are calculated from *n* = 12. Abbreviations: dClB = 1,4-dichlorobenzene: 0.1 ng/ml; mClB = chlorobenzene mix: 0.1 ng/ml; PU = phenuron: 10^−6^ M; MU = monuron: 10^−6^ M; DU = diuron: 10^−6^ M.

Figure 5 shows the PRL release in AdH cultures in the presence of the ED compounds tested. It can be seen that the ED compounds did not trigger relevant differences in PRL release (mean level ± SEM (ng PRL/mg protein): control: 7.13 ± 0.04; dClB 7.28 ± 0.03; mClB 7.26 ± 0.01; PU 7.12 ± 0.02; MU 7.02 ± 0.02; and DU 7.14 ± 0.01).

**Figure 6:**
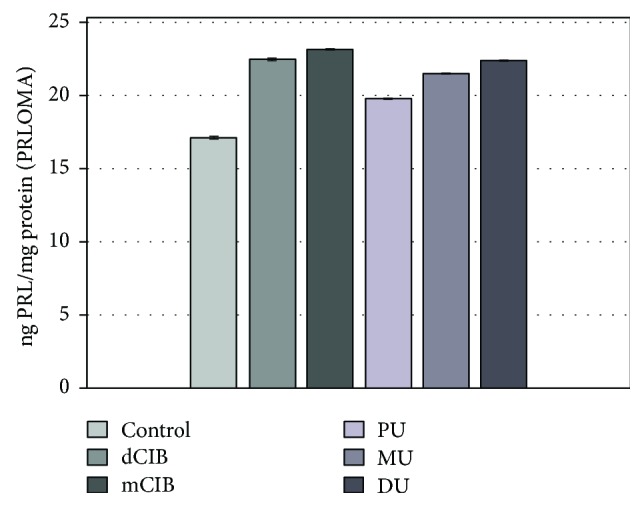
Effects of EDC on PRL release in rat PRLOMA cultures, *in vitro*. Mean (PRL/prolactin/level) ± SEM. The mean and SEM are calculated from *n* = 12. Abbreviations: dClB = 1,4-dichlorobenzene: 0.1 ng/ml; mClB = chlorobenzene mix: 0.1 ng/ml; PU = phenuron: 10^−6^ M; MU = monuron: 10^−6^ M; DU = diuron: 10^−6^ M. All EDC groups differ significantly from the control (*p* < 0.001) for basal release.

Figure 6 shows the PRL release of rat PRLOMA cultures in the presence of ED chemical agents as described in the experimental protocol. All the examined compounds showed a significant stimulating effect (dClB: 22.47 ± 0.03; mClB: 23.17 ± 0.02; PU: 19.82 ± 0.01; MU: 21.50 ± 0.02; and DU: 22.41 ± 0.02 ng PRL/mg protein) on the release of PRL compared to that of the control (17.14 ± 0.02 ng PRL/mg protein) in PRLOMA cultures.

## 4. Discussion and Conclusions

We have studied the effects of PU, MU, DU, dClB, and mClB as potential environmental factors, on the basal release of ACTH (Figure 3) (creative element: mobile network junction [49]), which plays a role in the essential functioning of the AdH/AC axis [30] (interpreted in the human homeostasis network as an “*AND*” function). The ED effects of the applied compounds could be detected, since each chemical agent generated significant changes in the ACTH secretion of PRLOMA cells (Figure 4) in the basal group. PRL release can be increased by enhancing peripheral estrogene levels, which can be the “*OR*” cycle in connection to ACTH release. In our work, we modeled a homeostatic regulatory neuroendocrine network both under normal regulation (AdH model) and under a neuroendocrine disturbance (+ER). (First, cell cycle change was induced via autoregulatory cell dysfunction [29].) In the PRLOMA cells that were already altered by the control cycle, the level of not only PRL (Figure 6), but also that of ACTH increased (Figure 4). The events of the neuroendocrine regulatory cycles in our investigation suggest that the outlined mechanisms can be regarded as homeostatic biological network elements [50].

The AVP-activated ACTH hormone secretion of AdH cells was significantly increased by all ED compounds used when compared to the control (Figure 3, +AVP group). ED agents caused increased ACTH release in the AVP-activated PRLOMA cells as well (Figure 4, +AVP group). It is well known that in biological complexities (biological networks), regulations appear as the resultants of activating and inhibiting functions [51]. Both in the normal AdH and PRLOMA cell types, the negative feedback effect of corticosterone was modeled by the inhibition of AVP-activated ACTH release [52]. Figure 3 demonstrates that AdH cells were able to maintain their inhibitory functions despite a treatment with EDC (Figure 3, +B+AVP group). Thus, the adaptive potential of healthy AdH cells in cases of chemical environmental load modeled by EDC remained reversibly regulated. The regulation of PRLOMA cells was incomplete in the presence of EDC (Figure 4, +B+AVP group); although the inhibitory effect of corticosterone on ACTH secretion is also present in PRLOMA, the maintenance of this effect is strongly disrupted by ED compounds (Figure 4). In this context, the results can be interpreted as an environmental disruption leading to irreversible processes. The presence of ED compounds enhanced the already overexpressed ACTH secretion in PRLOMA cells (Figure 4), which was further increased by AVP activation. Therefore, it can be assumed that both the structure and the endocrine cell function of PRLOMA were damaged [53]. Due to the role ACTH plays in essential life functions, this result may have significance in the environmental exposition of prolactinoma patients and in the development of adaptational potential disorders in healthy individuals [54], as ACTH regulation is present in human adaptation as an open dynamic requirement, which is modeled as an “*AND*” logical function algorithm. Our results show that PRL secretion in normal AdH cells was not influenced by EDC in the applied experimental system (Figure 5). However, the already elevated PRL secretion of PRLOMA cells was further enhanced by EDC (Figure 6).

HTP is defined in relation to dClB with a standard approach [43] and uniform risk assessment. The authors wished to provide an opportunity for comparison by following dClB effects too. Accordingly, uron/dClB relations were determined in AdH and PRLOMA cells. Our results show that the EDC classification of the examined uron compounds strongly approximates the effects of dClB in an AdH model (Figures 3 and 5). However, basal ACTH release of PRLOMA is more effective in the presence of DU (uron/dClB → 0.97–0.99) than dClB (+DU/dClB = 1.05). In baseline PRL secretion, the uron effects on AdH cells approximated those of dClB (urons/dClB: 0.96–0.98). In the case of PRLOMA, the EDC effects of the investigated uron compounds on PRL secretion approximated those of dClB (urons/dClB: 0.96–0.98). However, in the case of AVP-activated ACTH release in the PRLOMA model, dClB effect was exceeded by the agents MU and DU (PRLOMA: +AVP + MU/+AVP + dClB = 1.02; PRLOMA: +AVP + DU/+AVP + dClB = 1.03).

The HTP values of PRLOMA can be interpreted with double risk classification according to our present study, because when the doses of ED compounds exceed those necessary for irreversible effects (such as suspension of corticosterone inhibition), regulatory dysfunctions may present difficult-to-treat disease processes.

It can be seen from the above how important researching human homeostatic network disorders is when one wishes to evaluate the health consequences of environmental factors.

## Data Availability

Requests for data will be considered by the corresponding author.

## References

[1] Gavrilescu M., Demnerová K., Aamand J., Agathos S., Fava F. (2015). Emerging pollutants in the environment: present and future challenges in biomonitoring, ecological risks and bioremediation. *New Biotechnology*.

[2] Barber J. L., Sweetman A. J., van Wijk D., Jones K. C. (2005). Hexachlorobenzene in the global environment: emissions, levels, distribution, trends and processes. *Science of the Total Environment*.

[3] Holmes G. (2014). Australia’s pesticide environmental risk assessment failure: the case of diuron and sugarcane. *Marine Pollution Bulletin*.

[4] Bergman A., Heindel J. J., Jobling S., Kidd K. A., Zoeller R. T. (2013). *State of the Science of Endocrine Disrupting Chemicals - 2012: An Assessment of the State of the Science of Endocrine Disruptors Prepared by a Group of Experts for the United Nations Environment Programme (UNEP) and WHO*.

[5] Goodson W. H., Lowe L., Carpenter D. O. (2015). Assessing the carcinogenic potential of low-dose exposures to chemical mixtures in the environment: the challenge ahead. *Carcinogenesis*.

[6] Rajamani U., Gross A. R., Ocampo C., Andres A. M., Gottlieb R. A., Sareen D. (2017). Endocrine disruptors induce perturbations in endoplasmic reticulum and mitochondria of human pluripotent stem cell derivatives. *Nature Communications*.

[7] Santos-Silva A. P., Andrade M. N., Pereira-Rodrigues P. (2018). Frontiers in endocrine disruption: impacts of organotin on the hypothalamus-pituitary-thyroid axis. *Molecular and Cellular Endocrinology*.

[8] Schug T. T., Janesick A., Blumberg B., Heindel J. J. (2011). Endocrine disrupting chemicals and disease susceptibility. *The Journal of Steroid Biochemistry and Molecular Biology*.

[9] McLachlan J. A. (2001). Environmental signaling: what embryos and evolution teach us about endocrine disrupting chemicals. *Endocrine Reviews*.

[10] Mokarizadeh A., Faryabi M. R., Rezvanfar M. A., Abdollahi M. (2015). A comprehensive review of pesticides and the immune dysregulation: mechanisms, evidence and consequences. *Toxicology Mechanisms and Methods*.

[11] Nadal A., Quesada I., Tuduri E., Nogueiras R., Alonso-Magdalena P. (2017). Endocrine-disrupting chemicals and the regulation of energy balance. *Nature Reviews Endocrinology*.

[12] Preciados M., Yoo C., Roy D. (2016). Estrogenic endocrine disrupting chemicals influencing NRF1 regulated gene networks in the development of complex human brain diseases. *International Journal of Molecular Sciences*.

[13] López-Otín C., Blasco M. A., Partridge L., Serrano M., Kroemer G. (2013). The hallmarks of aging. *Cell*.

[14] Vicencio J. M., Galluzzi L., Tajeddine N. (2008). Senescence, apoptosis or autophagy? When a damaged cell must decide its path—a mini-review. *Gerontology*.

[15] Brace L. E., Vose S. C., Stanya K. (2016). Increased oxidative phosphorylation in response to acute and chronic DNA damage. *npj Aging and Mechanisms of Disease*.

[16] Kasanova Z., Hernaus D., Vaessen T. (2016). Early-life stress affects stress-related prefrontal dopamine activity in healthy adults, but not in individuals with psychotic disorder. *PLoS One*.

[17] Veitshans T., Klimov D., Thirumalai D. (1997). Protein folding kinetics: timescales, pathways and energy landscapes in terms of sequence-dependent properties. *Folding and Design*.

[18] Maslov S., Sneppen K. (2002). Specificity and stability in topology of protein networks. *Science*.

[19] Kotronoulas G., Stamatakis A., Stylianopoulou F. (2009). Hormones, hormonal agents, and neuropeptides involved in the neuroendocrine regulation of sleep in humans. *Hormones*.

[20] Cornejo M. P., Hentges S. T., Maliqueo M., Coirini H., Becu-Villalobos D., Elias C. F. (2016). Neuroendocrine regulation of metabolism. *Journal of Neuroendocrinology*.

[21] Nemeroff C. B. (2013). Psychoneuroimmunoendocrinology: the biological basis of mind-body physiology and pathophysiology. *Depression and Anxiety*.

[22] Papadimitriou A., Priftis K. N. (2009). Regulation of the hypothalamic-pituitary-adrenal axis. *Neuroimmunomodulation*.

[23] Wang R. S., Saadatpour A., Albert R. (2012). Boolean modeling in systems biology: an overview of methodology and applications. *Physical Biology*.

[24] Orton F., Lutz I., Kloas W., Routledge E. J. (2009). Endocrine disrupting effects of herbicides and pentachlorophenol: in vitro and in vivo evidence. *Environmental Science & Technology*.

[25] Valkusz Z., Nagyéri G., Radács M. (2011). Further analysis of behavioral and endocrine consequences of chronic exposure of male Wistar rats to subtoxic doses of endocrine disruptor chlorobenzenes. *Physiology & Behavior*.

[26] Oturan M. A., Edelahi M. C., Oturan N., El kacemi K., Aaron J. J. (2010). Kinetics of oxidative degradation/mineralization pathways of the phenylurea herbicides diuron, monuron and fenuron in water during application of the electro-Fenton process. *Applied Catalysis B: Environmental*.

[27] Motejlová H., Koci V. (2011). Application of 1,4-dichlorobenzene as a reference substance in the LCA methodology (life cycle assessment). *Acta Environmentalica Universitatis Comenianae (Bratislava)*.

[28] De Kloet E. R., Joels M., Holsboer F. (2005). Stress and the brain: from adaptation to disease. *Nature Reviews Neuroscience*.

[29] Sarkar D. K. (2006). Genesis of prolactinomas: studies using estrogen-treated animals. *Frontiers of Hormone Research*.

[30] Molnár Z., Pálföldi R., László A. (2015). Effects of chronic and subtoxic chlorobenzenes on adrenocorticotrophic hormone release. *Journal of Environmental Sciences*.

[31] Liu J., Krieger R. (2010). Phenylurea herbicides. *Hayes’ Handbook of Pesticide Toxicology*.

[32] Federico C., Pappalardo M., Leotta G. C., Minniti Z., Librando V., Saccone S., Kobayashi D., Watanabe E. (2014). Phenylurea herbicides: chemical properties and genotoxic effects. *Handbook on Herbicides*.

[33] Lintelmann J., Katayama A., Kurihara N., Shore L., Wenzel A. (2003). Endocrine disruptors in the environment (IUPAC technical report). *Pure and Applied Chemistry*.

[34] Lewis K. A., Tzilivakis J., Warner D. J., Green A. (2016). An international database for pesticide risk assessments and management. *Human and Ecological Risk Assessment: An International Journal*.

[35] Mnif W., Hassine A. I. H., Bouaziz A., Bartegi A., Thomas O., Roig B. (2011). Effect of endocrine disruptor pesticides: a review. *International Journal of Environmental Research and Public Health*.

[36] Kojima H., Katsura E., Takeuchi S., Niiyama K., Kobayashi K. (2004). Screening for estrogen and androgen receptor activities in 200 pesticides by in vitro reporter gene assays using Chinese hamster ovary cells. *Environmental Health Perspectives*.

[37] Morita M. (1977). Chlorinated benzenes in the environment. *Ecotoxicology and Environmental Safety*.

[38] Mackay D., Shiu W. Y., Ma K. C., Lee S. C. (2006). *Handbook of Physical-Chemical Properties and Environmental Fate for Organic Chemicals*.

[39] National Toxicology Program (1987). Toxicology and carcinogenesis studies of 1,4-dichlorobenzene (CAS no. 106-46-7) in F344/N rats and B6C3F1 mice (gavage studies). *National Toxicology Program Technical Report Series*.

[40] Randi A. S., Cocca C., Carbone V. (2006). Hexachlorobenzene is a tumor co-carcinogen and induces alterations in insulin-growth factors signaling pathway in the rat mammary gland. *Toxicological Sciences*.

[41] Ralph J. L., Orgebin-Crist M. C., Lareyre J. J., Nelson C. C. (2003). Disruption of androgen regulation in the prostate by the environmental contaminant hexachlorobenzene. *Environmental Health Perspectives*.

[42] den Besten C., Vet J. J. R. M., Besselink H. T. (1991). The liver, kidney, and thyroid toxicity of chlorinated benzenes. *Toxicology and Applied Pharmacology*.

[43] International Standard ISO 14044 (2006). Environmental management. Life cycle assessment. Requirements and guidelines. *International Organization for Standardization*.

[44] Molnár Z., Pálföldi R., László A. (2014). The effects of hypokalaemia on the hormone exocytosis in adenohypophysis and prolactinoma cell culture model systems. *Experimental and Clinical Endocrinology & Diabetes*.

[45] Lowry O. H., Rosebrough N. J., Farr A. L., Randall R. J. (1951). Protein measurement with the Folin phenol reagent. *The Journal of Biological Chemistry*.

[46] Brown H., Prescott R. (2006). *Applied Mixed Models in Medicine*.

[47] Singer J., Willett J. (2003). *Applied Longitudinal Data Analysis: Modeling Change and Event Occurrence*.

[48] SAS (2011). *SAS/STAT 9.3 User’s Guide*.

[49] Strand F. L., Rose K. J., King J. A., Segarra A. C., Zuccarelli L. A. (1989). ACTH modulation of nerve development and regeneration. *Progress in Neurobiology*.

[50] Gądek-Michalska A., Spyrka J., Rachwalska P., Tadeusz J., Bugajski J. (2013). Influence of chronic stress on brain corticosteroid receptors and HPA axis activity. *Pharmacological Reports*.

[51] McLachlan J. A. (2016). Environmental signaling: from environmental estrogens to endocrine-disrupting chemicals and beyond. *Andrology*.

[52] Jasnic N., Djordjevic J., Vujovic P., Lakic I., Djurasevic S., Cvijic G. (2013). The effect of vasopressin 1b receptor (V1bR) blockade on HPA axis activity in rats exposed to acute heat stress. *Journal of Experimental Biology*.

[53] Oh M. C., Aghi M. K. (2011). Dopamine agonist-resistant prolactinomas. *Journal of Neurosurgery*.

[54] Spencer R. L., Deak T. (2017). A users guide to HPA axis research. *Physiology and Behavior*.

